# Assessment of Self-Reported Factors Associated With Impaired Sleep in Hospitalized Adult Patients in Internal Medicine

**DOI:** 10.7759/cureus.25947

**Published:** 2022-06-15

**Authors:** Abdul Khalid, Rubina Rafique, Muhammad Arshad, Muzhar Hamdani

**Affiliations:** 1 Medicine, Azad Jammu and Kashmir Medical College, Muzaffarabad, PAK; 2 Integrative Medicine, Abbas Institute of Medical Sciences, Muzaffarabad, PAK

**Keywords:** sleep status, sleep pattern, hospital noise, sleep disturbance, sleep

## Abstract

Objectives

To evaluate the different personal, environmental, and social factors associated with the impaired sleep of patients who were admitted for treatment in the department of internal medicine in a tertiary care hospital in Azad Kashmir.

Material and methods

A single-center, exploratory, prospective study was conducted at Abbas Institute of Medical Sciences (AIMS), a public sector teaching hospital of Azad Jammu and Kashmir Medical College, Muzaffarabad, between November 2021 and January 2022. A standard Performa was used to collect and document the demographic information, the duration and quality of sleep at home and after admission to the hospital. The patients were asked to identify and report the most important factors contributing to disturbed sleep in the hospital. Statistical analysis was performed using SPSS version 23.0 (SPSS Inc., Chicago, IL, USA), continuous parametric variables were reported as mean ± standard deviation; nonparametric continuous variables were reported as median; and categorical variables were expressed as percentages. Exploratory factor analysis was performed and principal components were extracted.

Results

As reported by the patients, the main factors contributing to disturbed sleep were underlying medical conditions, noise, overcrowding and gathering of people, pain, fever, lighting, weather conditions, and medical interventions. Exploratory component factor analysis showed significant loading of overcrowding and lighting in the ward on components 1 and 2. Component 3 was loaded with underlying illness, noise, pain and fever, uncomfortable mattress, and treatment interventions by the medical staff.

Conclusions

There were many personal and environmental factors, which contributed towards impaired sleep in hospitalized patients. The underlying medical conditions, noise, overcrowding, lighting, pain and fever, are the most commonly reported factors by hospitalized patients.

## Introduction

Good quality sleep of adequate duration is necessary for a healthy life. However, the ideal amount of sleep required each night varies among different individuals. The recommended sleep duration for healthy adults (26-64 years) is seven to nine hours daily [1,2]. The deprivation of night sleep is associated with a number of physical and mental health conditions, which include lack of energy, increased fatigue, daytime sleepiness, low mood, and poor functioning capacity [3].

Insufficient and disturbed sleep is common in hospitalized patients due to underlying medical conditions and numerous environmental and exogenous factors [4]. The deprivation of sleep is associated with increased discomfort for patients, delayed recovery, and increased morbidity. Several studies have shown that insomnia leads to irritability, fatigue, and aggressive behavior in hospitalized patients [5].

The environmental and exogenous conditions are mostly related to the particular type of indoor facility, social behavior, and cultural norms of the residents. There is wide diversity in these social norms and behaviors, not only in different societies in different countries but also in different parts of the same country. There are highly connected family systems and traditions in Pakistan and Azad Kashmir. The overcrowding of medical units with attendants of the admitted patients is a common scene in public hospitals. Most of the public sector hospitals are not fully air-conditioned and weather conditions are often very harsh in different parts of the country. As there is no available research on this topic from this region, this study was planned to evaluate the different personal, environmental, and social factors associated with the disturbed sleep of patients who were admitted for treatment in the department of internal medicine in a tertiary care hospital in Azad Kashmir.

## Materials and methods

This single-center, exploratory, prospective study was conducted at Abbas Institute of Medical Sciences (AIMS), a public sector teaching hospital of Azad Jammu and Kashmir Medical College, Muzaffarabad, between November 2021 and January 2022. The study was approved by the Institutional Ethical Review Committee of the Abbas Institute of Medical Sciences (ERC/AIMS/24-2021/6). All adult patients admitted to medical units (one and two) of the department of internal medicine were included in the study. The institutional ethical review committee of the Abbas Institute of Medical Sciences approved the study. A standard Performa was developed and used to collect and document the demographic information, the duration and quality of sleep at home and after admission to the hospital. The patients were asked to identify and report the most important factors contributing to disturbed sleep in the hospital. Inclusion and exclusion criteria are mentioned in Table 1.

**Table 1:** Inclusion and exclusion criteria.

Inclusion criteria	Exclusion criteria
All adult, indoor patients from internal medicine who consented to participate	Patients are admitted to intensive care and high dependency areas.
	History of sleep disorder before admission.
	Patients are already on hypnotics, anxiolytics, or antidepressants.

Statistical analysis

All statistical analyses were performed using SPSS version 23.0 (SPSS Inc., Chicago, IL, USA). For all tests, p values of <0.05 were considered statistically significant. Continuous parametric variables were reported as mean ± standard deviation; nonparametric continuous variables were reported as median and categorical variables were expressed as percentages. Exploratory factor analysis was performed and principal components were extracted.

## Results

A total of 100 adults and indoor patients (53% male and 47% female) were enrolled in the study. About 45% of the patients were suffering from some chronic underlying medical condition (Table 2), while 55% were admitted with some acute medical illness.

**Table 2:** Baseline characteristics of patients. COPD: chronic obstructive pulmonary disease.

Gender	Male%	Female%
	53	47
Mean sleep time at night	Home (hours)	Hospital (hours)
	6.32 SD ± 2.20	5.24 SD ± 1.86
Self-reported satisfaction with quality and duration of sleep		
Sleep quality and duration	Home (% of patients)	Hospital (% of patients)
Normal	57	46
Less	31	47
Good	12	7
Day time sleeping	39	42
Co-morbid medical conditions		
Medical conditions	Percentage of patients (%)	
Hypertension	20	
Diabetes mellitus	9	
Ischemic heart disease	5	
Chronic asthma/COPD	11	

The mean sleep time at home was 6.32 (SD ± 2.20) hours at night. The mean sleep time in the hospital was 5.24 (SD ± 1.85) at night. On average, participants were sleeping 1.08 hours less in the hospital. The subjective satisfaction of the participants with the duration and quality of sleep at home and in hospital is also shown in Table 2. The difference was statistically significant (p<0.001) when compared with a one-sample t-test (Table 3).

**Table 3:** One-sample test.

	t	df	Sig. (two-tailed)	95% Confidence interval of the difference	
				Lower	Upper
Sleep at home	28.65	99	0.001	5.88	6.76
Sleeping time at night in hospital	28.04	99	0.001	8.65	9.97

The main factors, as reported by the patients, contributing to disturbed sleep were underlying medical conditions, noise, over-crowding and gathering of people, pain, fever, lighting, weather conditions, and medical interventions. The percentage of patients reporting these factors is shown in Table 4.

**Table 4:** Factors associated with sleep disturbance.

Factors	% of patients
Due to sickness	40
Noise	34
Gathering of peoples	28
Pain	22
Fever	20
Light	16
Uncomfortable mattress	10
Cold and humidity	6
Staff treatment (injections, drips)	6
Insects	1

The exploratory factor analysis showed the correlation of these factors as shown in Table 5. There was a statistically significant strong co-relation among all these factors contributing to disturbed sleep in these patients.

**Table 5:** Exploratory factor analysis.

Correlation	Noise in ward	Light in ward	Overcrowding	Mattress	Weather	Pain	Fever	Illness	Insects	Injections or medical procedures
Noise in the ward	1.00	0.32	0.117	0.18	0.08	0.23	0.169	−0.06	0.14	−0.09
Light in ward	0.32	1.00	0.396	−0.05	0.11	0.09	−0.014	−0.07	−0.04	−0.11
Overcrowding	0.11	0.39	1.00	−0.05	0.03	0.15	0.022	−0.05	−0.06	0.03
Mattress	0.18	−0.05	−0.059	1.00	0.19	0.06	0.083	−0.06	0.30	0.05
Weather	0.08	0.11	0.030	0.19	1.0	−0.03	−0.021	0.13	0.39	0.11
Pain	0.23	0.09	0.153	0.06	−0.03	1.00	0.881	0.45	−0.05	0.17
Fever	0.16	−0.01	0.022	0.08	−0.02	0.88	1.000	0.40	−0.05	0.08
Illness	−0.06	−0.07	−0.055	−0.06	0.13	0.45	0.408	1.00	0.12	0.22
Insects	0.14	−0.04	−0.063	0.30	0.39	−0.05	−0.050	0.12	1.00	0.39
Injections or medical procedures	−0.09	−0.11	0.030	0.05	0.11	0.17	0.084	0.22	0.39	1.00

There was significant loading of overcrowding and lighting in the ward on components 1 and 2. Component 3 was loaded with underlying illness, noise, pain and fever, uncomfortable mattress, and treatment interventions by the medical staff.

## Discussion

A good quality sleep of adequate duration is essential for health and especially for the well-being of patients [6]. It is important to recognize modifiable barriers to good quality sleep in a particular health setting where patients are being treated. There are wide variations in these factors in different societies and cultures and even in the same society, depending upon the health care facility and type of care and nature of underlying conditions of the patients [7]. Hence, it is imperative that every health care setting and its different departments have knowledge and awareness of all the factors adversely affecting the sleep of admitted patients and adopt strategies to improve the sleep quality of the patients. This study was designed to evaluate different personal and environmental factors contributing to disturbed sleep in patients who were admitted to the department of internal medicine at this institute. It has to be mandatory that clinicians should have clear insight and knowledge of these factors in their clinical domain to plan specific interventions to be tailored to individual patients to optimize the duration and quality of sleep.

The most important and frequently reported personal factor contributing towards disturbed sleep (40% of the participants) was related to the underlying medical condition of the patients. It was followed by the noise (34%), gathering of people and overcrowding by the attendants of the patients (28%), pain and fever (22% and 20%), the lighting in the ward (16%), an uncomfortable mattress (10%), environmental factors (6%), interventions by medical staff (6%), and insects (1%). It is apparent that the sleep of most of the patients was adversely affected by modifiable factors. The exploratory principal factor analysis also confirmed the significant loading of environmental factors contributing to disturbed sleep. There was a statistically significant correlation among these factors (Table 5). The overcrowding due to visitors and attendants of the admitted patients was the significant factor contributing to disturbed sleep. The scene of the gathering of family members who often surround the admitted patient and sit and share the hospital bed may be a rare scenario in the west but a common daily routine in our social setup, both disturbing the sleep and management of patients by medical staff.

A study by Dobing and colleagues from Canada reported the most reasons for poor sleep as noise (59%), nursing interruptions (30%), uncomfortable beds (18%), bright lights (16%), unfamiliar surroundings (14%), and pain (9%) [8]. The results of that study differed from the present study in the extent of medical interventions (30% and 6%, respectively). This could be explained by some patients who reported from far-flung areas were admitted for observation in our medical units, and little intervention was needed for that subset.

In a descriptive study by Missildine and colleagues in elderly hospitalized patients, they found that “noise and light” were the most frequently related environmental factors responsible for disturbed sleep [9]. These findings were similar to the findings in our study where underlying illness, followed by noise and lighting, were the most frequently reported reasons for disturbed sleep. It provides reasonable guidance to plan sleep cycle optimizing strategies that could be generally adopted (noise reductions and controlling light) for the indoor facility and others that would target the personal factors (underlying illness, pain and fever). Clinician should give attention and importance to sleep hygiene, while they are preoccupied with the medical conditions, comorbidities, clinical evaluation and management of the hospitalized patients.

A cross-sectional study of 2005 patients in the Netherlands by Wesselius demonstrated that the duration and quality of sleep in hospitalized patients were significantly affected by many potentially modifiable hospital-related factors [10]. They found noise from other patients, sounds of medical devices, pain, and toilet visits as the most common and reported sleep-disturbing factors. There were some similarities with our study and some significant differences as well. These differences highlight the importance of societal diversity and contextual considerations in contributing towards disturbed sleep.

Another study from Thailand revealed a high prevalence of poor sleep quality in hospitalized patients, where light exposure and pain were demonstrated to be the factors associated with poor sleep quality [11]. These findings also support the common contribution of environmental and personal factors leading to poor sleep in hospitalized patients.

A cross-sectional study by D Souza in a tertiary care hospital explored different factors influencing the sleep of the admitted patients [12]. They found the number of roommates, type of the ward, sleep medication, hospitalization period, and severity of pain as the main contributors to poor sleep. About 69% of the patients had poor sleep in that study. It was inferred that adequate management of pain and modification of the ward interior and atmosphere could improve the sleep quality of inpatients.

Another study demonstrated multiple factors, common and often under-recognized, responsible for sleep disruption in hospitalized patients, which also included environmental noise [13]. The findings in this study also support the findings of our study. Environmental noise has been found in several studies as the most common factor responsible for sleep interruptions [14,15]. The fact remains that it is difficult to find the ideal amount of sleep for every patient in different medical settings who is suffering from a particular disease and has comorbidities. There are a number of common factors associated with and contributing towards disturbed sleep. There are several adverse effects of sleep deprivation [16,17]. If not addressed properly, it will further compromise the well-being of patients. There are many tools available to quantify sleep and can be used in a clinical setting to specifically gauge the sleep adequacy of hospitalized patients [18]. The clinicians need to explore, identify, and rectify the contextual modifiable factors contributing to disturbed sleep in the setting of their clinical domain.

The major limitations of the study were strong confounding contextual factors related to the environment of a specific medical unit, local norms and social behaviors. Hence, the generalizability of the results is limited. However, at the same time, this is also a strength of the study as these sleep-disturbing factors are needed to be explored in different settings.

## Conclusions

There were a number of personal and environmental factors that contributed towards impaired sleep in hospitalized patients. The underlying medical conditions, noise, overcrowding, lighting, pain and fever being the most commonly reported factors by hospitalized patients. These are common, easily recognizable sleep-impacting factors, but often ignored in medical units. Clinicians should recognize and modify these factors in their clinical domain and environment. The good quality of sleep of hospitalized patients should be given the same priority as the clinical evaluation and management of underlying medical conditions of the patients. Clinicians in medical units should review periodically and take the necessary steps for the prevention of factors contributing towards impaired adequacy and quality of sleep, which have proven negative associations with health care outcomes.
